# Supplementary material for: Focus on Polish nurses’ health condition: a cross-sectional study
Source: PeerJ. 2022 Mar 21;10:e13065. doi: 10.7717/peerj.13065 (PMC8944332; doi:10.7717/peerj.13065)
Supplement: Supplemental Information 2 [file peerj-10-13065-s002.pdf]

□□□

SURVEY QUESTIONNAIRE – NURSES' HEALTH CONDITION

Socio-demogrphic data of survey nurses:

Age: ....

Place of residence:

- ❑ City
- ❑ Vilage

Place of work:

- ❑ Hospital
- ❑ Ambulatory

Work system:

- ❑ One shift work
- ❑ Shift work and night duty

Education:

- ❑ Medical secondary education
- ❑ Bechelor's degree
- ❑ Master's degree

Additional qualification:

- ❑ No
- ❑ Yes

Self-assessment of health condition:

- ❑ Very good
- ❑ Good
- ❑ I have no opinion
- ❑ Bad

Participation in preventive examinations:

- ❑ No
- ❑ Yes
